# PET with Different Radiopharmaceuticals in Neuroendocrine Neoplasms: An Umbrella Review of Published Meta-Analyses

**DOI:** 10.3390/cancers13205172

**Published:** 2021-10-15

**Authors:** Giorgio Treglia, Ramin Sadeghi, Francesco Giovinazzo, Federica Galiandro, Salvatore Annunziata, Barbara Muoio, Alexander Stephan Kroiss

**Affiliations:** 1 Clinic of Nuclear Medicine, Imaging Institute of Southern Switzerland, Ente Ospedaliero Cantonale, 6500 Bellinzona, Switzerland; 2 Department of Nuclear Medicine and Molecular Imaging, Lausanne University Hospital, 1011 Lausanne, Switzerland; 3 Academic Education, Research and Innovation Area, General Directorate, Ente Ospedaliero Cantonale, 6500 Bellinzona, Switzerland; 4 Faculty of Biology and Medicine, University of Lausanne, 1011 Lausanne, Switzerland; 5 Faculty of Biomedical Sciences, Università della Svizzera italiana, 6900 Lugano, Switzerland; 6 Nuclear Medicine Research Center, Mashhad University of Medical Sciences, Mashhad 9919991766, Iran; sadeghir@mums.ac.ir; 7 Dipartimento di Scienze Mediche e Chirurgiche, Fondazione Policlinico Universitario A. Gemelli IRCCS, 00168 Rome, Italy; francesco.giovinazzo@policlinicogemelli.it (F.G.); federica.galiandro@aulss3.veneto.it (F.G.); 8 UOC Medicina Nucleare, TracerGLab, Dipartimento di Diagnostica per Immagini, Radioterapia Oncologica ed Ematologia, Fondazione Policlinico Universitario A. Gemelli IRCCS, 00168 Rome, Italy; salvatore.annunziata@policlinicogemelli.it; 9 Department of Medicine and Oncology, Institute of Southern Switzerland, Ente Ospedaliero Cantonale, 6500 Bellinzona, Switzerland; barbara.muoio@eoc.ch; 10 Department of Nuclear Medicine, Medical University Innsbruck, 6020 Innsbruck, Austria; alexander.kroiss@i-med.ac.at

**Keywords:** PET, positron emission tomography, neuroendocrine, meta-analysis, umbrella review, diagnostic performance, prognosis, impact

## Abstract

**Simple Summary:**

Functional imaging methods and, in particular, positron emission tomography (PET) using several radiopharmaceuticals may play a pivotal role in patients with neuroendocrine neoplasms including neuroendocrine tumors (NETs) located in different sites, paraganglioma (PGL) and neuroblastoma (NB), recurrent medullary thyroid carcinoma (rMTC) and aggressive neuroendocrine neoplasms. Several radiopharmaceuticals can be used in this setting such as Gallium-68 somatostatin analogues (^68^Ga-SSA), Fluorine-18 fluorodihydroxyphenylalanine (^18^F-FDOPA), Gallium-68 exendin-4 (^68^Ga-exendin-4), Fluorine-18 fluorodeoxyglucose (^18^F-FDG). This umbrella review provides an evidence-based summary about meta-analyses on diagnostic performance, prognostic value, clinical impact and safety of PET with different radiopharmaceuticals in patients with neuroendocrine neoplasms. Overall, evidence-based data support the use of PET with different radiopharmaceuticals in patients with neuroendocrine neoplasms but with specific indications for each radiopharmaceutical.

**Abstract:**

Background: Several meta-analyses have reported quantitative data about the diagnostic performance, the prognostic value, the impact on management and the safety of positron emission tomography (PET) including related hybrid modalities (PET/CT or PET/MRI) using different radiopharmaceuticals in patients with neuroendocrine neoplasms. We performed an umbrella review of published meta-analyses to provide an evidence-based summary. Methods: A comprehensive literature search of meta-analyses listed in PubMed/MEDLINE and Cochrane Library databases was carried out (last search date: 30 June 2021). Results: Thirty-four published meta-analyses were selected and summarized. About the diagnostic performance: ^68^Ga-SSA PET yields high diagnostic performance in patients with NETs and PGL; ^18^F-FDOPA PET yields good diagnostic performance in patients with intestinal NETs, PGL, NB, being the best available PET method in detecting rMTC; ^68^Ga-exendin-4 PET has good diagnostic accuracy in detecting insulinomas; ^18^F-FDG PET has good diagnostic performance in detecting aggressive neuroendocrine neoplasms. About the prognostic value: ^68^Ga-SSA PET has a recognized prognostic value in well-differentiated NETs, whereas ^18^F-FDG PET has a recognized prognostic value in aggressive neuroendocrine neoplasms. A significant clinical impact of ^68^Ga-SSA PET and related hybrid modalities in patients with NETs was demonstrated. There are no major toxicities or safety issues related to the use of PET radiopharmaceuticals in patients with neuroendocrine neoplasms. Conclusions: Evidence-based data support the use of PET with different radiopharmaceuticals in patients with neuroendocrine neoplasms with specific indications for each radiopharmaceutical.

## 1. Introduction

Neuroendocrine neoplasms are described in almost every tissue, either in the pure endocrine organs or in the so-called diffuse neuroendocrine system. Evidence indicates that neuroendocrine neoplasia may be well or poorly differentiated, with diverse incidence and prevalence in different organs. It has been proposed that well-differentiated neuroendocrine neoplasms are universally defined as neuroendocrine tumors (NETs) and poorly differentiated neuroendocrine neoplasms as neuroendocrine carcinomas (NECs) [1,2].

Imaging methods play a pivotal role in detection, characterization, and therapy planning in patients with NETs and NECs. In particular, functional imaging techniques as positron emission tomography (PET) and related hybrid imaging modalities (that combine functional and morphological information) as positron emission tomography/computed tomography (PET/CT) and positron emission tomography/magnetic resonance imaging (PET/MRI) are increasingly used in patients with NETs and NECs [3].

Several PET radiopharmaceuticals evaluating different metabolic pathways or receptor status can be used to assess these tumors, including somatostatin analogs radiolabeled with Gallium-68 or Copper-64 (^68^Ga-SSA or ^64^Cu-SSA) evaluating the somatostatin receptor (SSR) expression, Fluorine-18 fluorodihydroxyphenylalanine (^18^F-FDOPA) assessing the uptake, decarboxylation and storage of amine precursors, Fluorine-18 fluorodeoxyglucose (^18^F-FDG) evaluating the glucose metabolism, and ^68^Ga-exendin-4 an agonist of the glucagon-like protein-1 receptor (GLP-1R) [4,5]. Beyond the diagnostic information, PET with different radiopharmaceuticals was also used to obtain prognostic information in patients with neuroendocrine neoplasms [6].

As several meta-analyses about the role of PET with different radiopharmaceuticals in NETs and NECs have been published so far, we aimed to perform an evidence-based synthesis. This umbrella review was carried out to summarize evidence-based data and in particular quantitative measures about the diagnostic performance, the prognostic value, the clinical impact and the safety of PET and related hybrid imaging modalities in patients with neuroendocrine neoplasms.

## 2. Materials and Methods

This umbrella review of published meta-analyses was performed according to a predefined protocol [7]. The objective of this manuscript is to summarize quantitative data of published meta-analyses about PET in neuroendocrine neoplasm. Therefore, a search string was created using a combination of keywords and Boolean operators. The complete search algorithm used for the literature search (including the different keywords used) is reported in Appendix A at the end of this manuscript.

After the creation of the search string, a comprehensive literature search of PubMed/MEDLINE and Cochrane Library databases was performed to find published meta-analyses on PET and related hybrid modalities (PET/CT or PET/MRI) using different radiopharmaceuticals in patients with neuroendocrine neoplasms. The literature search was updated until 30 June 2021. No language restrictions nor time limits were used.

About the inclusion criteria, meta-analyses investigating the diagnostic performance, the prognostic value, the impact on management and the safety of PET by using different radiopharmaceuticals in patients with NETs and NECs were eligible for inclusion. Only systematic reviews with statistical analyses (meta-analyses) were included.

Titles and abstracts of the retrieved meta-analyses were reviewed, full-text articles were downloaded and checked in detail. Lastly, the eligible meta-analyses were selected applying the inclusion criteria mentioned above.

For each selected meta-analysis, information was collected about the indication of PET imaging, PET radiopharmaceuticals evaluated, authors, year of publication, number of original articles and patients included in each meta-analysis, pooled outcome measures with 95% confidence interval values (95% CI), statistical heterogeneity and publication bias. The pooled outcome measures collected for diagnostic performance were detection rate (DR), sensitivity, specificity, and area under the receiving–operating characteristics (ROC) curve. The pooled outcome measures collected for the prognostic studies were expressed as hazard ratios (HR) whereas the clinical impact was evaluated as the pooled percentage of change in management.

Quality assessment of the included meta-analyses was also performed according to the specific quality assessment tool developed by the Center of Evidence-Based Medicine of the University of Oxford.

To minimize possible biases, two review authors independently performed the literature search, the selection of studies according to the inclusion criteria, the data extraction and the quality assessment of included meta-analyses. Disagreements were discussed through an online consensus meeting.

## 3. Results

From the comprehensive computer literature search from PubMed/MEDLINE and Cochrane Library databases, 185 records were retrieved and 34 meta-analyses were selected according to the predefined inclusion and exclusion criteria [8,9,10,11,12,13,14,15,16,17,18,19,20,21,22,23,24,25,26,27,28,29,30,31,32,33,34,35,36,37,38,39,40,41]. These meta-analyses were published in the last ten years. Quality assessment of included meta-analyses is available in Supplementary Material (Table S1). Some examples of the application of different PET radiopharmaceuticals to detect neuroendocrine neoplasms are shown in the Supplemental Material (Figure S1). The main characteristics of selected meta-analyses are presented in Table 1, Table 2 and Table 3 and summarized as follows. We followed a hierarchical order in the description of the results of PET in neuroendocrine neoplasms according to this priority: (a) outcome measure (e.g., diagnostic performance, prognostic value, clinical impact, safety); (b) type of neuroendocrine neoplasm evaluated by PET imaging; (c) type of PET radiopharmaceuticals used.

### 3.1. Diagnostic Performance of PET with Different Radiopharmaceuticals in Neuroendocrine Neoplasms

Overall, 26 meta-analyses containing information on the diagnostic performance of PET with different radiopharmaceuticals in patients with NETs and NECs were included (Table 1) [10,11,13,14,15,16,18,19,21,22,23,24,25,28,29,30,31,32,33,34,35,36,37,38,39,40]. These evidence-based manuscripts provided quantitative data about the diagnostic performance of PET and related hybrid modalities (PET/CT and PET/MRI) using different radiopharmaceuticals in patients with suspected NETs [31], thoracic and/or gastroenteropancreatic NETs [10,14,15,18,23,28,29,30,34,40], small cell lung cancer (SCLC) [24], metastatic NETs with unknown primary (CUP-NETs) [13,25], paraganglioma (PGL) [16,19,29,35], neuroblastoma (NB) [22,32,39], recurrent medullary thyroid carcinoma (rMTC) [11,21,29,33,36,38] and Merkel cell carcinoma (MCC) [37].

#### 3.1.1. Suspected NETs

One meta-analysis assessed the diagnostic performance of ^68^Ga-SSA PET or PET/CT in the initial diagnosis of NETs. Patients were examined by PET or PET/CT for suspected NETs based on clinical features, elevated levels of biochemical markers, conventional imaging suggestive of NETs, or a combination of these conditions. The ^68^Ga-SSA PET or PET/CT were very accurate in this setting with a per-patient-based pooled sensitivity and specificity of 91% and 94%, respectively [31].

#### 3.1.2. Thoracic and/or Gastroenteropancreatic NETs

Several meta-analyses evaluated the diagnostic performance of PET or PET/CT with different radiopharmaceuticals in patients with thoracic and/or gastroenteropancreatic NETs [10,14,15,18,23,28,29,30,34,40].

About ^68^Ga-SSA PET or PET/CT, the first meta-analysis by Treglia et al. demonstrated that these methods were very accurate in detecting thoracic and/or gastroenteropancreatic NETs with a pooled sensitivity and specificity of 93% and 91%, respectively, on a per-patient-based analysis [34]. These findings were confirmed by an updated meta-analysis by Geijer et al. reporting a pooled sensitivity and specificity of 93% and 96%, respectively, on a per-patient-based analysis [15]. Yang et al. performed a meta-analysis comparing PET with two different SSA (DOTATOC and DOTATATE) in NETs: the pooled sensitivity of ^68^Ga-DOTATOC and ^68^Ga-DOTATATE PET in the diagnosis of NETs, calculated on a per-patient-based analysis, was 93% and 96%, respectively, and the pooled specificity was 85% and 100%, respectively, with an overlap of 95%CI values among these PET methods [40]. Another meta-analysis by Deppen et al. focused on ^68^Ga-DOTATATE PET or PET/CT confirmed the high pooled sensitivity (90.9%) and specificity (90.6%) of these methods in detecting thoracic and gastroenteropancreatic NETs [14]. As recently reported by Liu et al., the patient-based pooled sensitivities for ^68^Ga-SSA PET or PET/CT in general, and for ^68^Ga-DOTATOC, ^68^Ga-DOTATATE, and ^68^Ga-DOTANOC PET or PET/CT in particular, were 92%, 95%, 92% and 87%, respectively, whereas the pooled patient-based specificities were 91%, 91%, 88% and 90%, respectively. Lesion-based sensitivity and specificity of ^68^Ga-SSA PET or PET/CT were 95% and 93%, respectively [23]. Interestingly, the pooled sensitivities of ^68^Ga-SSA PET/CT were 92%, 90% and 58% in the grade 1 (G1), grade 2 (G2) and grade 3 (G3) subgroups of NETs, respectively [23].

^18^F-FDOPA PET or PET/CT were also used for assessing thoracic and gastroenteropancreatic NETs and the meta-analysis of Rufini et al. reported a per-patient-based pooled sensitivity and specificity of 77% and 95%, respectively, in this setting [29].

^18^F-FDG PET or PET/CT provided a lower accuracy in this setting with a per-patient-based pooled sensitivity of 70%. Interestingly, the pooled sensitivities of ^18^F-FDG PET/CT were 38%, 55% and 71% in the G1, G2 and G3 subgroups of NETs, respectively [23].

Some meta-analyses were focused on specific locations of thoracic and gastroenteropancreatic NETs [10,18,28,30].

Bauckneht et al. calculated that the pooled sensitivity and specificity of ^68^Ga-SSA PET or PET/CT for the assessment of primary pancreatic NETs were 79.6% and 95%, respectively. Pooled detection rates for the primary pancreatic tumors were 81% and 92%, respectively, at patient-based and lesion-based analysis [10].

In the specific subgroup of insulinomas, radiolabelled exendin-4 imaging showed excellent results, superior to other PET modalities. The meta-analysis of Shah et al. reported high pooled sensitivity (94%) and specificity (83%) of ^68^Ga-exendin-4 PET/CT for insulinoma localization; these values were significantly higher compared to radiolabeled exendin-4 single-photon emission computed tomography (SPECT)/CT [30]. Recently, Piccardo et al. compared the diagnostic performance of ^68^Ga-SSA and ^18^F-FDOPA PET/CT in the group of patients with intestinal NETs through a head-to-head meta-analytic approach. The pooled sensitivity of ^18^F-FDOPA PET/CT was 83% and 95% on a per-patient-based analysis and per-lesion-based analysis, respectively. The ^68^Ga-SSA PET/CT showed a pooled sensitivity of 88% and 82% on a per-patient-based analysis and per-lesion-based analysis, respectively. No significant differences were found between the two PET radiopharmaceuticals on a per-patient-based analysis; conversely, a trend towards significance in favor of ^18^F-FDOPA PET/CT was identified on a per-lesion-based analysis [28].

About pulmonary carcinoids, Jiang et al. performed a meta-analysis to compare the diagnostic performance of ^68^Ga-SSA and ^18^F-FDG PET/CT in this setting. The pooled sensitivity of ^68^Ga-SSA and ^18^F-FDG PET/CT in the detection of pulmonary carcinoid were 90% and 71%, respectively. Interestingly, typical carcinoids revealed apparently higher uptake on ^68^Ga-SSA PET/CT compared with atypical carcinoids and the ratios of SUVmax on ^68^Ga-SSA PET/CT to that on ^18^F-FDG PET/CT were significantly higher in typical carcinoids than atypical carcinoids [18].

#### 3.1.3. Small Cell Lung Cancer (SCLC)

Lu et al. demonstrated through their meta-analysis that ^18^F-FDG PET or PET/CT are valuable imaging tools for the pretherapeutic assessment of extensive disease (ED) in patients with SCLC, an aggressive neuroendocrine neoplasm. The pooled estimates of sensitivity and specificity of ^18^F-FDG PET or PET/CT for the detection of ED in SCLC were 97.5% and 98.2%, respectively [24].

#### 3.1.4. Metastatic NETs with Unknow Primary Tumor (CUP-NETs)

^68^Ga-SSA PET/CT is highly effective in locating the primary and metastatic sites of CUP-NETs according to available meta-analyses [13,25]. The detection rate of ^68^Ga-SSA PET/CT in detecting primary NETs in patients with metastatic disease on a per-patient-based analysis was 56% as reported by De Dosso et al. [13]. Another meta-analysis on the same topic showed a similar value of detection rate (61%); the pooled sensitivity and specificity of ^68^Ga-SSA PET/CT in identifying CUP-NETs were 82% and 55%, respectively [25]. The most frequent sites of primary unknown NETs detected by ^68^Ga-SSA PET/CT were the bowel and the pancreas [13,25]. The histological grade of primary NETs detected by ^68^Ga-SSA PET/CT were G1 in 56.5% of cases; G2 in 27.6% of cases and G3 in 9.8% of cases [25]. The most common metastatic sites were the liver (57.9%), followed by lymph nodes (22.8%) and bones (12.8%) [25].

#### 3.1.5. Paragangliomas (PGL)

The diagnostic performance of PET and related hybrid modalities using different PET radiopharmaceuticals in detecting PGL was evaluated by several meta-analyses [16,19,29,35].

Kan et al. compared the diagnostic performance of ^68^Ga-SSA and ^18^F-FDG PET in PGL and reported a pooled sensitivity and specificity of 95% and 87%, respectively, for 68Ga-SSA PET; the same estimated were 85% and 55%, respectively, for ^18^F-FDG PET. In PGL patients with germline mutations, the diagnostic sensitivities of ^68^Ga-SSA and ^18^F-FDG PET were 97% and 79%, respectively [19]. About ^68^Ga-SSA PET or PET/CT, Han et al. reported a pooled per-lesion detection rate of 93%, significantly higher than that of ^18^F-FDOPA PET (80%), ^18^F-FDG PET (74%) or radioiodinated metaiodobenzylguanidine (^123^/^131^I-MIBG) scintigraphy (38%). A greater proportion of head and neck PGL was significantly associated with higher detection rates of ^68^Ga-SSA PET, whereas other variables, including multifocal or metastatic disease, germline mutation status, sporadic type, catecholamine-secretory PGLs, age, and tumor size, were not significant in meta-regression analyses [16].

A meta-analysis focused on ^8^F-FDOPA PET or PET/CT in PGL reported a pooled sensitivity on a per-patient- and on a per-lesion-based analysis of 91% and 79%, respectively. The pooled specificity of ^18^F-FDOPA PET or PET/CT calculated on a per-patient- and per-lesion-based analysis was 95% and 95%, respectively. Interestingly, a significant increase in sensitivity of ^18^F-FDOPA PET or PET/CT (95% on a per-patient-based analysis and 91% on a per-lesion-based analysis) was observed when patients with succinate dehydrogenase subunit B (SDHB) gene mutations were excluded from the analysis [35]. The high diagnostic performance of ^18^F-FDOPA PET or PET/CT in patients with PGL was also described in the meta-analysis of Rufini et al. reporting a pooled patient-based sensitivity and specificity of 92% and 92%, respectively [29].

#### 3.1.6. Neuroblastoma (NB)

Some meta-analyses assessed the diagnostic performance of PET or PET/CT in patients with NB [22,32,39].

According to the meta-analysis of Xia et al., PET or PET/CT with ^18^F-FDG or ^18^F-FDOPA showed higher accuracy than ^123^/^131^I-MIBG on a per-lesion-based analysis and might be the preferred modality for the staging of NB [39]. The high diagnostic accuracy of ^18^F-FDG or ^18^F-FDOPA PET or PET/CT in NB was also confirmed by the meta-analysis of Li et al. reporting a global sensitivity and specificity of 91% and 78%, respectively [22]. Unfortunately, in these meta-analyses, data on ^18^F-FDG and ^18^F-FDOPA PET were pooled together.

In detecting bone metastases and bone marrow involvement in pediatric NB the pooled sensitivity and specificity of ^18^F-FDG PET or PET/CT were 87% and 96%, respectively [32].

#### 3.1.7. Recurrent Medullary Thyroid Carcinoma (rMTC)

Several studies have evaluated the diagnostic performance of PET and PET/CT using ^68^Ga-SSA, ^18^F-FDOPA and ^18^F-FDG in patients with rMTC, reporting different detection rates [11,21,29,33,36,38].

The diagnostic performance of ^68^Ga-SSA PET or PET/CT in rMTC is lower compared to that of the same imaging method in the majority of NETs. A meta-analysis showed a per-patient-based detection rate of 63.5% in rMTC. This detection rate increased in rMTC patients with higher serum calcitonin levels (83% for calcitonin >500 ng/L) [38].

About ^18^F-FDOPA PET or PET/CT in rMTC, the detection rate on a per-patient- and per-lesion-based analysis were 66% and 71%, respectively. The detection rate significantly increased in patients with serum calcitonin >1000 ng/L (86%) and calcitonin doubling times <24 months (86%) [33].

About ^18^F-FDG PET or PET/CT in rMTC, the detection rate on a per-patient-based analysis was 59%. This value increased in patients with serum calcitonin ≥1000 ng/L (75%), CEA ≥5 ng/mL (69%), calcitonin doubling times < 12 months (76%), and CEA doubling times <24 months (91%) [36]. The pooled sensitivities of ^18^F-FDG PET and PET/CT were 68% and 69%, respectively [11].

A recent network meta-analysis indicated that, compared to other PET methods, ^18^F-FDOPA PET showed the best performance for the detection of rMTC in both patient- and lesion-based analyses regardless of serum calcitonin or CEA levels and calcitonin doubling time [21].

#### 3.1.8. Merkel Cell Carcinoma (MCC)

In patients with MCC, an aggressive neuroendocrine neoplasm, ^18^F-FDG PET or PET/CT demonstrated high pooled sensitivity (90%) and specificity (98%), being accurate imaging methods in this setting [37].

#### 3.1.9. Multiple Endocrine Neoplasia (MEN) Syndromes and Ectopic Cushing Syndrome (ECS)

There are no published meta-analyses on different PET radiopharmaceuticals in patients with MEN syndromes or ECS. However, the literature data seem to suggest a relevant diagnostic role of PET imaging in this setting. In particular, the latest evidence has shown that ^68^Ga-SSA PET provides good diagnostic accuracy in detecting MEN syndromes-related NETs higher than that of SSR scintigraphy. The ^18^F-FDOPA PET seems to have a potential role in detecting MEN-2-related NETs, whereas ^18^F-FDG PET is potentially useful in identifying aggressive NETs with poorer outcomes [42]. The tumor causing ECS due to adrenocorticotropic hormone (ACTH) hypersecretion is often difficult to localize on conventional imaging methods. A systematic review of the literature demonstrated that ^68^Ga-SSA PET has better sensitivity in the diagnosis of bronchial carcinoids causing ECS and ^18^F-FDG PET appears superior for SCLC and other aggressive tumors causing ECS [43].

### 3.2. Prognostic Value of PET with Different Radiopharmaceuticals in Neuroendocrine Neoplasm

Overall, six meta-analyses containing information on the prognostic value of PET with different radiopharmaceuticals in patients with NETs and NECs were included (Table 2) [8,12,17,20,27,41]. These evidence-based manuscripts provided quantitative data about the prognostic value of ^68^Ga-SSA PET or ^18^F-FDG PET in patients with thoracic and/or gastroenteropancreatic NETs [8,17,20] or SCLC [12,27,41] in terms of disease control rate (DCR), event-free survival (EFS), progression-free survival (PFS) or overall survival (OS).

#### 3.2.1. Thoracic and/or Gastroenteropancreatic NETs

About ^68^Ga-SSA PET, a meta-analysis by Lee et al. demonstrated that uptake of ^68^Ga-SSA at PET expressed as maximal standardized uptake value (SUVmax), could be used as an objective prognosis predictor. Low SUVmax at ^68^Ga-SSTR PET was associated with a worse prognosis for PFS and OS in patients with thoracic and/or gastroenteropancreatic NETs. In well-differentiated NETs, ^68^Ga-SSA PET had more prognostic value compared with all grades of NETs [20].

About ^18^F-FDG PET, two different meta-analyses demonstrated that ^18^F-FDG PET has a significant prognostic value in patients with thoracic and/or gastroenteropancreatic NETs. The ^18^F-FDG PET might be a useful prognostic biomarker in conjunction with the histologic grade and can help select the optimal treatment strategy in patients with NETs [8,17]. Alevroudis et al. demonstrated that ^18^F-FDG PET prior to peptide receptor radionuclide therapy (PRRT) appears to be a useful tool in patients with NETs to predict tumor response and survival outcomes and a negative ^18^F-FDG PET before PRRT is associated with prolonged PFS and OS [8]. Han et al. reported that ^18^F-FDG uptake was a significant prognostic factor in terms of EFS and OS for thoracic and/or gastroenteropancreatic NETs: higher ^18^F-FDG uptake was associated with a worse prognosis for EFS and OS in patients with thoracic and/or gastroenteropancreatic NETs. Greater prevalence of grade 3 NETs was associated with a higher prognostic value of ^18^F-FDG PET [17].

#### 3.2.2. Small Cell Lung Cancer (SCLC)

Three different meta-analyses clearly demonstrated that ^18^F-FDG PET has a significant prognostic value in patients with SCLC, in particular taking into account metabolic parameters at PET such as SUVmax of the primary lesion, metabolic tumor volume (MTV) or total lesion glycolysis (TLG) [12,27,41]. Higher values of these metabolic parameters were associated with a poorer prognosis regarding EFS, PFS and OS [12,27,41].

### 3.3. Clinical Impact of PET with Different Radiopharmaceuticals in Neuroendocrine Neoplasms

Four meta-analyses evaluated the clinical impact of PET with different radiopharmaceuticals in patients with neuroendocrine neoplasms, including thoracic and/or gastroenteropancreatic NETs, CUP-NETs and SCLC [9,13,26,31] (Table 3).

#### 3.3.1. Thoracic and/or Gastroenteropancreatic NETs and CUP-NETs

A meta-analysis by Barrio et al. demonstrated that ^68^Ga-SSA PET/CT findings resulted in management changes in 44% of the patients with thoracic and/or gastroenteropancreatic NETs. Studies on implemented management reported change in 44% of cases, studies on intended management in 41% of patients. In patients performing ^68^Ga-SSA PET/CT in addition to a prior SSR scintigraphy, 39% of patients experienced a change in treatment strategy. About the type of management change (inter-modality versus intra-modality), inter-modality changes occurred more than three times more frequently than intra-modality changes [9]. Another meta-analysis on the same topic confirmed these findings reporting a change in management by using ^68^Ga-SSA PET/CT in 45% of NETs, with the majority of the changes involving surgical planning and patient selection for PRRT [31].

The pooled percentage of change in management by using ^68^Ga-SSA PET/CT in CUP-NETs was 20% [13].

#### 3.3.2. SCLC

In SCLC patients the change of binary staging (upstaging from limited to extensive SCLC or downstaging from extensive to limited SCLC) is associated with a change in management. The pooled percentage of change of binary SCLC staging using ^18^F-FDG PET/CT in SCLC was 15% according to the meta-analysis of Martucci et al. [26].

### 3.4. Safety of PET with Different Radiopharmaceuticals in Neuroendocrine Neoplasms

Report of harm possibly related to PET with different radiopharmaceuticals in neuroendocrine neoplasms was rare; no study reported major toxicity or safety issues [14].

## 4. Discussion

We performed an umbrella review of published meta-analyses on PET and related hybrid imaging modalities in neuroendocrine neoplasms to summarize the quantitative data provided by these evidence-based manuscripts. As a matter of fact, umbrella reviews represent the highest level of evidence synthesis currently available and are becoming increasingly influential in biomedical literature [7]. The most characteristic feature of umbrella review is that this type of evidence synthesis only considers for inclusion the highest level of evidence and in particular systematic reviews and meta-analyses [7]. In particular, we were interested to summarize quantitative estimates of diagnostic performance, prognostic value, clinical impact and safety of PET with different radiopharmaceuticals in neuroendocrine neoplasms taking into account published meta-analyses. Another advantage of an umbrella review is to clarify which are the fields of interest that need more evidence-based data [7].

Based on the results provided by this umbrella review, there are suggestive evidence-based data to support the following statements about PET with different radiopharmaceuticals described in the available meta-analyses:

(A) Diagnostic performance:
  - ^68^Ga-SSA PET and related hybrid modalities yield high diagnostic performance in patients with suspected NETs, and in detecting thoracic and gastroenteropacreatic NETs, CUP-NETs and PGL.
  - ^18^F-FDOPA PET and related hybrid modalities yield good diagnostic performance in patients with intestinal NETs, PGL, NB and can be a good alternative to other PET methods in these settings; ^18^F-FDOPA is the best PET radiopharmaceutical in detecting rMTC even if the detection rate is suboptimal.
  - ^68^Ga-exendin-4 PET has good diagnostic accuracy in detecting insulinomas and it could be used as the preferred PET method in this setting.
  - ^18^F-FDG PET and related hybrid modalities yield good diagnostic performance in detecting aggressive neuroendocrine neoplasms (e.g., high-grade NETs, SCLC, NB and MCC).
(B) Prognostic value:
  - The ^68^Ga-SSA PET has a recognized prognostic value in well-differentiated NETs; in these patients, a lower uptake at ^68^Ga-SSA PET is associated with a worse prognosis.
  - The ^18^F-FDG PET has a recognized prognostic value in aggressive neuroendocrine neoplasms; in these patients, higher values of metabolic parameters are associated with a worse prognosis.
(C) Clinical impact:
  - There is a significant clinical impact of ^68^Ga-SSA PET and related hybrid modalities in patients with NETs.
(D) Safety:
  - There are no major toxicities or safety issues related to the use of PET radiopharmaceuticals in patients with neuroendocrine neoplasms.

Awareness of the results described in this umbrella review may affect patient care by providing supportive evidence for more effective use of PET and related hybrid modalities with different radiopharmaceuticals in patients with neuroendocrine neoplasms. Overall, the main findings of this umbrella review seem to support the currently available guidelines [44,45,46,47] and consensus statements on PET in neuroendocrine neoplasms [48].

According to health technology assessment (HTA) principles which are valid also for PET imaging [49,50], several factors should be taken into account to support the usefulness of a diagnostic imaging method (as PET, PET/CT or PET/MRI with different radiopharmaceuticals) including availability, diagnostic performance, clinical effectiveness, safety, legal, organization and economic aspects [49,50]. We focused our umbrella review on diagnostic performance, prognostic value, clinical impact and safety of PET with different radiopharmaceuticals because these data were provided by available meta-analyses. On the other hand, we should underline that the reduced availability of some PET radiopharmaceuticals worldwide may represent an important limitation hampering their use in current clinical practice.

Another aspect that is crucial for the correct introduction and use of an imaging method in the clinical scenario is its cost-effectiveness. Even if the cost-effectiveness of PET imaging in neuroendocrine neoplasms was not evaluated by specific meta-analyses, a recent study comparing ^68^Ga-SSA PET/CT, SSR SPECT/CT and CT underlined that the use of ^68^Ga-SSA PET/CT in NETs is cost-effective. The additional costs of ^68^Ga-SSA PET/CT, when compared to CT alone, are justified in the light of potential savings in therapy costs and better outcomes [51].

Some limitations of the meta-analyses included in this umbrella review should be underlined because they could hamper the achievement of definitive conclusions on the role of PET with different radiopharmaceuticals in patients with neuroendocrine neoplasms. First of all, in some selected meta-analyses a limited number of original studies was included; this could have influenced the statistical power of the meta-analysis. Significant statistical heterogeneity was found in several meta-analyses about the outcome measures described; this potential bias could be due to differences among the included studies in terms of characteristics of patients included, methods, quality, reference standard and study design [52,53]. Furthermore, publication bias was reported in some meta-analyses pointing out that studies with significant findings were more likely to be published than those reporting non-significant results [52,53]. Overall, included meta-analyses were heterogeneous and biases cannot be excluded. However, we made efforts to minimize possible biases in the selection of these articles: two review authors independently performed the literature search, the selection of studies according to the inclusion criteria, the data extraction and the quality assessment of included meta-analyses.

In this umbrella review, we did not consider the use of PET with different radiopharmaceuticals in patients with congenital hyperinsulinism (CHI) or oncogenic osteomalacia (OO) because these conditions are not caused by neuroendocrine neoplasms sensu stricto. However, we should underline that several meta-analyses have demonstrated that ^18^F-FDOPA PET/CT has high sensitivity and specificity in differentiating between the focal and diffuse form of CHI and in localizing the focal form of CHI [54,55,56], whereas in detecting culprit tumors causing OO ^68^Ga-SSA PET/CT has better sensitivity than other PET and SPECT methods and should be used as first-line imaging modality [57,58,59].

As a suggestion for further research, it would be interesting to obtain quantitative data through a meta-analytic process about the use of PET imaging with different radiopharmaceuticals in assessing treatment response in patients with neuroendocrine neoplasms as currently there are no meta-analyses published on this topic. Furthermore, some meta-analyses selected in this umbrella review (in particular those including fewer studies) could be updated including more studies and increasing their statistical power.

## 5. Conclusions

Evidence-based data support the use of PET with different radiopharmaceuticals in patients with neuroendocrine neoplasms with specific indications for each radiopharmaceutical.

## Figures and Tables

**Table 1:** Diagnostic performance of PET with different radiopharmaceuticals in neuroendocrine neoplasm.

Disease	PET Tracer	Ref.	Year	Studies (pts) Included in the Meta-Analysis	Pooled DR (95% CI)	Pooled Sensitivity (95% CI)	Pooled Specificity (95% CI)	AUC (95% CI)	Statistical Heterogeneity	Publication Bias
Suspected NETs	^68^Ga-SSA	[31]	2018	6 (598)	NR	pb: 91% (85–94)	pb: 94% (86–98)	NR	NR	No
Thoracic and GEP-NETs	^68^Ga-SSA	[34]	2012	16 (567)	NR	pb: 93% (91–95)	pb: 91% (82–97)	pb: 0.96	Yes	NR
		[15]	2013	22 (2105)	NR	pb: 93% (91–94)	pb: 96% (95–98)	pb: 0.98 (0.95–1)	Yes	No
		[40]	2014	10 (416)	NR	pb ^a^: 93% (89–96) pb ^b^: 96% (91–99)	pb ^a^: 85% (74–93) pb ^b^: 100% (82–100)	pb ^a^: 0.96 pb ^b^: 0.98	Yes	NR
		[14]	2016	17 (971)	NR	pb: 91% (81–96)	pb: 91% (78–96)	NR	Yes	No
		[23]	2020	30 (3401)	NR	pb: 92% (89–95) lb: 95% (86–98)	pb: 91% (83–95) lb: 93% (83–97)	pb: 0.96 (0.94–0.98) lb: 0.98 (0.96–0.99)	Yes	Yes
	^18^F-FDOPA	[29]	2013	8 (289)	NR	pb: 77% (71–82)	pb: 95% (87–98)	pb: 0.94	Yes	NR
	^18^F-FDG	[23]	2020	30 (3401)	NR	pb: 70% (41–89)	pb: 97% (70–100)	pb: 0.94 (0.92–0.96)	Yes	No
P-NETs	^68^Ga-SSA	[10]	2020	18 (NR)	pb: 81% (65–90) lb: 92% (80–97)	pb: 80% (71–87)	pb: 95% (75–100)	NR	Yes	Yes
Insulinomas	^68^Ga-exendin-4	[30]	2021	16 (179)	NR	lb: 94% (NR)	lb: 83% (NR)	NR	NR	NR
I-NETs	^68^Ga-SSA	[28]	2021	6 (112)	NR	pb: 88% (79–94) lb: 82% (63–94)	NR	NR	Yes	No
	^18^F-FDOPA	[28]	2021	6 (112)	NR	pb: 83% (70–92) lb: 95% (89–99)	NR	NR	Yes	No
L-NETs	^68^Ga-SSA	[18]	2019	14 (352)	NR	pb: 90% (82–95)	NR	NR	Yes	NR
	^18^F-FDG	[18]	2019	14 (352)	NR	pb: 71% (66–76)	NR	NR	Yes	NR
ED-SCLC	^18^F-FDG	[24]	2014	12 (369)	NR	pb: 97% (94–99)	pb: 98% (95–100)	pb: 0.98	No	NR
CUP-NETs	^68^Ga-SSA	[13]	2019	12 (383)	pb: 56% (48–63)	NR	NR	NR	Yes	No
		[25]	2020	10 (484)	pb: 61% (53–69)	pb: 82% (63–92)	pb: 55% (31–77)	pb: 0.69 (0.65–0.73)	Yes	No
PGL	^68^Ga-SSA	[19]	2018	17 (629)	NR	pb: 95% (92–97) lb: 96% (93–98)	pb: 87% (63–96)	pb: 0.88 (0.85–0.91)	Yes	No
		[16]	2019	9 (215)	lb: 93% (91–95)	NR	NR	NR	No	Yes
	^18^F-FDOPA	[35]	2012	11 (275)	NR	pb: 91% (87–94) lb: 79% (76–81)	pb: 95% (86–99) lb: 95% (84–99)	pb: 0.95 lb: 0.94	Yes	NR
		[29]	2013	13 (401)	NR	pb: 92% (88–95)	pb: 92% (85–97)	pb: 0.95	Yes	NR
		[16]	2019	9 (215)	lb: 80% (69–88)	NR	NR	NR	No	Yes
	^18^F-FDG	[19]	2018	17 (629)	NR	pb: 85% (78–91) lb: 83% (68–92)	pb: 55% (37–73)	pb: 0.78 (0.74–0.81)	Yes	No
		[16]	2019	9 (215)	lb: 74% (46–91)	NR	NR	NR	No	Yes
NB	^18^F-FDG	[32]	2021	7 (127)	NR	pb: 78% (64–88) lb: 89% (81–94)	pb: 90% (79–97) lb: 78% (70–85)	pb: 0.94	Yes	No
	Several	[39]	2017	11 (1081)	NR	pb: 82% (75–88) lb: 90% (88–92)	pb: 70% (52–84) lb: 71% (63–79)	pb: 0.69 lb: 0.94	No	No
		[22]	2020	11 (580)	NR	pb: 91% (86–94)	pb: 78% (66–86)	pb: 0.93 (0.90–0.95)	Yes	No
rMTC	^68^Ga-SSA	[38]	2017	9 (152)	pb: 63% (49–77)	NR	NR	NR	Yes	NR
	^18^F-FDOPA	[33]	2012	8 (146)	pb: 66% (58–74) lb: 71% (67–75)	NR	NR	NR	Yes	NR
		[29]	2013	8 (161)	NR	pb: 62% (54–69)	NR	NR	Yes	NR
	^18^F-FDG	[36]	2012	24 (538)	pb: 59% (54–63)	NR	NR	NR	Yes	NR
		[11]	2012	15 (815)	NR	pb: 69% (64–74)	NR	NR	Yes	NR
	Several	[21]	2020	14 (306)	NR	NR	NR	NR	Yes	No
MCC	^18^F-FDG	[37]	2013	6 (92)	NR	pb: 90% (80–96)	pb: 98% (90–100)	pb: 0.96	No	No

Abbreviations: ^a^ = DOTATOC; ^b^ = DOTATATE; ^18^F-FDG = Fluorine-18 fluorodeoxyglucose; ^18^F-FDOPA = Fluorine-18 fluorodihydroxyphenylalanine; ^68^Ga-exendin-4 = Gallium-68 exendin-4; ^68^Ga-SSA = Gallium-68 somatostatin analogues; 95% CI = 95% confidence interval; AUC = area under the receiver operating characteristic curve; CUP-NETs = unknown primary neuroendocrine tumors; DR = detection rate; ED-SCLC = extensive disease—small cell lung cancer; GEP-NETs = gastroenteropancreatic neuroendocrine tumors; I-NETs = intestinal neuroendocrine tumors; L-NETs = lung carcinoids; lb = lesion-based; MCC = Merkel cell carcinoma; NB = neuroblastoma; NETs = neuroendocrine tumors; NR = not reported; pb = patient-based; PGL = paragangliomas; PET = positron emission tomography; P-NETs = pancreatic neuroendocrine tumors; pts = patients; rMTC = recurrent medullary thyroid carcinoma.

**Table 2:** Prognostic value of PET with different radiopharmaceuticals in neuroendocrine neoplasms.

Disease	PET Tracer	Ref.	Year	Studies (pts) Included in the Meta-Analysis	Variable Assessed for Prognosis	Pooled OR for DCR (95% CI)	Pooled HR for PFS/EFS (95% CI)	Pooled HR for OS (95% CI)	Statistical Heterogeneity	Publication Bias
Thoracic and GEP-NETs	^68^Ga-SSA	[20]	2019	8 (474)	SUV_max_	NR	2.31 (1.34–4.0)	2.97 (1.71–5.15)	Yes	Yes
	^18^F-FDG	[8]	2021	12 (1492)	PET results (+/−) before PRRT	4.85 (2.27–10.36)	2.45 (1.48–4.04)	2.25 (1.55–3.28)	Yes	Yes
		[17]	2021	23 (1799)	uptake (high/low)	NR	2.84 (2.21–3.64)	3.50 (2.75–4.45)	Yes	Yes
SCLC	^18^F-FDG	[41]	2018	12 (1062)	metabolic parameters (SUV_max_)	NR	1.09 (1.02–1.17)	1.13 (1.05–1.22)	No	No
		[27]	2019	7 (680)	metabolic parameters (MTV, TLG)	NR	MTV: 2.78 (1.39–5.53)	MTV: 2.42 (1.46–4.03)TLG: 1.61(1.24–2.07)	Yes	NR
		[12]	2021	19 (NR)	metabolic parameters (SUV_max_, MTV)	NR	SUV_max_: 1.24 (0.94–1.63) MTV: 3.22 (1.96–5.28)	SUV_max_: 1.50 (1.17–1.91) MTV: 2.83 (2.00–4.01)	Yes	Yes

Abbreviations: ^18^F-FDG = Fluorine-18 fluorodeoxyglucose; ^68^Ga-SSA = Gallium-68 somatostatin analogues; 95% CI = 95% confidence interval; +/− = positive/negative; DCR = disease control rate; EFS = event-free survival; GEP-NETs = gastroenteropancreatic neuroendocrine tumors; HR = hazard ratio; MTV = metabolic tumor volume; NR = not reported; OS = overall survival; PET = positron emission tomography; pts = patients; PFS = progression-free survival; PRRT = peptide receptor radionuclide therapy; SCLC = small cell lung cancer; SUV_max_ = maximal standardized uptake value; TLG = total lesion glycolysis.

**Table 3:** Clinical impact of PET with different radiopharmaceuticals in neuroendocrine neoplasms.

Disease	PET Tracer	Ref.	Year	Studies (pts) Included in the Meta-Analysis	Pooled Change of Management (95% CI)	Statistical Heterogeneity	Publication Bias
Thoracic and GEP-NETs	^68^Ga-SSA	[9]	2017	14 (1561)	44% (36–51)	Yes	NR
		[31]	2018	9 (NR)	45% (36–55)	Yes	NR
CUP-NETs	^68^Ga-SSA	[13]	2019	4 (114)	20% (9–33)	Yes	No
SCLC	^18^F-FDG	[26]	2020	6 (277)	15% (9–21)	Yes	No

Abbreviations: ^18^F-FDG = Fluorine-18 fluorodeoxyglucose; ^68^Ga-SSA = Gallium-68 somatostatin analogues; 95% CI = 95% confidence interval; CUP-NETs = unknown primary neuroendocrine tumors; GEP-NETs = gastroenteropancreatic neuroendocrine tumors; NR = not reported; PET = positron emission tomography; pts = patients; SCLC = small cell lung cancer.

## Supplementary Materials

The following are available online at https://www.mdpi.com/article/10.3390/cancers13205172/s1, Table S1. Quality assessment of the included meta-analyses. Figure S1. Case examples of the application of PET with different radiopharmaceuticals to detect neuroendocrine neoplasms.

## Appendix A

Search string used for the literature search: ((PET) OR (positron)) AND (meta-analysis) AND ((neuroendocrine) OR (NET) OR (NEN) OR (NEC) OR (medullary thyroid) OR (MTC) OR (carcinoid*) OR (small cell) OR (large cell) OR (SCLC) OR (LCNEC) OR (GEP) OR (insulinoma*) OR (hyperinsulinism) OR (gastrinoma*) OR (VIPoma*) OR (somatostatinoma*) OR (islet cell) OR (pancreatic endocrine) OR (pheochromocytoma*) OR (paraganglioma*) OR (PGL) OR (Pheo) OR (neuroblastoma*) OR (Merkel) OR (multiple endocrine) OR (MEN1) OR (MEN2) OR (von Hippel-Lindau) OR (VHL) OR (neurofibromatosis) OR (tuberous sclerosis) OR (Carney)).
