# Supplementary material for: PET with Different Radiopharmaceuticals in Neuroendocrine Neoplasms: An Umbrella Review of Published Meta-Analyses
Source: Cancers (Basel). 2021 Oct 15;13(20):5172. doi: 10.3390/cancers13205172 (PMC8533943; doi:10.3390/cancers13205172)
Supplement: Supplementary file 1 [file cancers-13-05172-s001.zip › cancers-1381291-supplementary.pdf]

cancers

MDPI

Supplementary Materials: PET with Different Radiopharmaceuticals in Neuroendocrine Neoplasms: An Umbrella Review of Published Meta-Analyses

Table S1. Quality assessment of the included meta-analyses.

Study	PICO question	Search strategy	Inclusion criteria	Quality assessment of included studies	Heterogeneity and methods to address the heterogeneity	Publication bias evaluation
Alevroudis et al. [8]	Impact of ^{18}F-FDG PET status on disease control rate, progression-free survival (PFS), and overall survival (OS) in neuroendocrine tumors (NETs) patients receiving peptide receptor radionuclide therapy (PRRT)	Medline, Embase, Cochrane Library, and Web of Science databases for published and unpublished reports (conference abstracts) in any language	Retrospective and prospective single- and multi-center cohort studies on patients with NETs receiving PRRT. Disease control rate according to RECIST or SWOG criteria, PFS and OS following PRRT should be reported. Surgical series with a sample size of at least 10 NETs patients undergoing PRRT	The Newcastle-Ottawa scale (NOS) template was used for quality/risk of bias assessment	I^2 index was used. No method was used to address the cause of heterogeneity	Evaluated by funnel plots and Egger's test
Barrio et al. [9]	Impact of somatostatin receptor (SSTR) PET/CT on the management of patients with NETs	It seems inadequate: only PubMed was searched. No data on language of articles	Original research and cohort studies reporting change in management after somatostatin receptor imaging; number of cases ≥ 10	No quality assessment available	I^2 index was used. Heterogeneity was further explored using subgroup analyses	No publication bias evaluation available
Bauckneht et al. [10]	Diagnostic performance of SSTR PET/CT for the detection of primary lesion and initial staging of pancreatic NETs	PubMed, Scopus, Embase, and Google Scholar databases were searched. Only English language articles	Diagnostic studies with a sample size > 10 cases and adequate diagnostic indices	QUADAS-2 was used for quality assessment	I^2 index was used. No method to address the cause of heterogeneity	Funnel plots were used
Cheng et al. [11]	Diagnostic performance of ^{18}F-FDG-PET and PET/CT for detection of recurrent or metastatic medullary thyroid carcinoma (MTC)	MEDLINE and EMBASE were searched. Only English language studies	Diagnostic studies with a sample size > 6 cases and adequate diagnostic indices	Quality was evaluated for each included study	Only Cochrane Q test was reported. Subgroup analyses were used to address the heterogeneity cause	No data available
Christensen et al. [12]	Prognostic value of one or several ^{18}F-FDG PET parameters in patients with small cell lung cancer	A search was performed in PubMed, Cochrane Library, and Embase. Only English language studies	Studies of baseline or post treatment ^{18}F-FDG PET providing hazard ratio (HR) and 95% confidence intervals (95%CI) for PFS or OS or sufficient data to extract HR and 95%CI	Quality in Prognostic Studies (QUIPS) tool was used. In the "study confounding" domain, the covariates stage, age, and sex were assessed	I^2 index and Cochrane Q value were used. No method was used to address the cause of heterogeneity	Funnel plots were used
De Dosso et al. [13]	Detection rate (DR) of SSTR PET/CT in patients with metastatic NETs and unknown primary tumors (CUP)	PubMed/MEDLINE, EMBASE, and Cochrane library databases were searched. No language restriction	Studies or subsets of studies investigating the DR of CUP by using SSTR PET/CT in patients with metastatic NET histologically proved; sample size > 10 cases	QUADAS-2 was used	I^2 index and Cochrane Q values. Heterogeneity was further explored by subgroup analyses	Funnel plots and Egger's test
Deppen et al. [14]	^{68}Ga-DOTATATE PET safety and efficacy compared to somatostatin receptor scintigraphy and conventional imaging for pulmonary and gastroenteropancreatic NETs	Medline, EMBASE, Web of Science and Cochrane Reviews electronic databases were searched. No language restriction	Primary trials or studies with >10 cases conducted to investigate diagnosis for pulmonary or gastroenteropancreatic NETs	QUADAS	I^2 index and Cochrane Q values. Heterogeneity was further explored by subgroup analyses	Funnel plots and Deek's funnel plot asymmetry test

Geijer et al. [15]	Diagnostic accuracy of SSTR PET in patients with known or suspected NETs	The databases PubMed/MEDLINE and Embase were searched. Only limited to 1 November 2011 to 31 December 2012 (update)	Studies on SSTR PET or PET/CT performed in patients with NETs in the thorax or abdomen; and sample size of at least 8 patients.	QUADAS-2 modified by removing the question "If a threshold was used, was it prespecified?"	I^2 index and Cochrane Q values. Threshold analysis using sROC. Sensitivity analysis was used to explore the heterogeneity	Funnel plots
Han et al. [16]	Performance of SSTR PET in the detection of pheochromocytomas and paragangliomas	PubMed and Embase databases without language restriction	Original articles with sample size larger than 5 cases of pheochromocytomas and paragangliomas assessed by SSTR PET	QUADAS-2	I^2 test was used. Heterogeneity was explored by subgroup analyses and meta-regression	Funnel plot and Egger's test
Han et al. [17]	Prognostic value of ^{18}F-FDG PET for NETs	PubMed and Embase databases without language restriction	Original articles reporting OS, PFS or other quantitative prognostic indices for ^{18}F-FDG PET in NETs	Quality in Prognostic Studies tool	I^2 test was used. Heterogeneity was explored by subgroup analyses and meta-regression	Funnel plot, Egger's test, and trim and fill method
Jiang et al. [18]	Diagnostic accuracy and prediction efficiency of histological subtypes of ^{18}F-FDG and SSTR PET in primary pulmonary carcinoids	PubMed, EMBASE, and Web of Science without language or date limitation	Original articles with enough data to extract sensitivity for ^{18}F-FDG or SSTR PET in pulmonary carcinoids	QUADAS-2	I^2 index was used; sROC was used to explore threshold effect	No data available
Kan et al. [19]	Diagnostic performance of ^{18}F-FDG and SSTR PET in metastatic pheochromocytomas and paragangliomas	PubMed/MEDLINE, ScienceDirect and Web of Science without language or date limitation	No specific section in the study	QUADAS-2	I^2 index and Cochrane Q values. Threshold analysis using sROC. Subgroup analyses were done to explore the cause of heterogeneity	Deeks' funnel plot asymmetry test
Lee et al. [20]	Prognostic value of the maximum standardized uptake value (SUVmax) of SSTR PET in patients with NETs	PubMed, Scopus, Cochrane, and Embase were searched	Studies that reported survival data regarding PFS and/or OS, and studies that included SUVmax of SSTR PET as a prognostic parameter in NETs	A predefined quality assessment scale	I^2 index and Cochrane Q values. Subgroup analyses were done to explore the cause of heterogeneity	Funnel plots and Egger's test
Lee et al. [21]	Network meta-analysis (NMA)using direct comparison studies with 2 or more PET radiopharmaceuticals for detection of recurrent medullary thyroid carcinoma (MTC)	English-language literature searches of PubMed and EMBASE databases	Diagnostic tests on the PET or PET/CT imaging methods including 2 or more PET radiopharmaceuticals in the same subjects to compare the DR of recurrent MTC	QUADAS-2	I^2 index and Cochrane Q values. Subgroup analyses were done to explore the cause of heterogeneity	Funnel plots
Li et al. [22]	Diagnostic accuracy of PET(CT) in patients with neuroblastoma	The Cochrane, PubMed, and Embase database were searched	Diagnostic studies on PET in neuroblastoma. The type of the radiotracer is not mentioned	Nothing provided	I^2 index and Cochrane Q values. Threshold analysis using sROC. Subgroup analyses were done to explore the cause of heterogeneity	Deeks' funnel asymmetry plot
Liu et al. [23]	To compare the diagnostic value of SSTR and ^{18}F-FDG PET or PET/CT in NETs	MEDLINE, PubMed, Embase and Cochrane Library databases for English-language articles	Diagnostic studies on SSTR or ^{18}F-FDG PET in NETs if provided enough data to calculate diagnostic indices	QUADAS-2	I^2 index and Cochrane Q values. Threshold analysis using sROC. Subgroup analyses and meta-regression were done to explore the cause of heterogeneity	Funnel plots and Egger's test
Lu et al [24]	Diagnostic accuracy of ^{18}F-FDG PET or PET/CT in	PubMed/MEDLINE and EMBASE	Diagnostic studies on ^{18}F-FDG PET in SCLC with enough	A predefined checklist was	I^2 index and Cochrane Q values.	No data

	the pretherapeutic staging of patients with small-cell lung cancer (SCLC)	with no language restriction	data to calculate diagnostic indices	used for quality assessment	Threshold analysis using sROC. No method was used to further explore the heterogeneity	
Ma et al. [25]	Diagnostic accuracy of SSTR PET for carcinoma unknown primary (CUP) NETs	PubMed/MEDLINE and ScienceDirect with no language restriction	Diagnostic studies on accuracy of SSTR PET for carcinoma unknown primary (CUP) NETs	QUADAS-2	I^2 index and Cochrane Q values. Threshold analysis using sROC. Subgroup analyses and meta-regression were done to explore the cause of heterogeneity	Deeks' asymmetry test and funnel plots
Martucci et al. [26]	Impact of ^{18}F-FDG PET/CT in staging SCLC	PubMed/MEDLINE, EMBASE, and Cochrane library with no language restriction	All original studies in which the main outcome was the change of binary SCLC staging using ^{18}F-FDG PET/CT or reported the diagnostic accuracy of ^{18}F-FDG PET/CT staging compared to conventional staging in SCLC and the impact of ^{18}F-FDG PET/CT staging on survival of SCLC patients	QUADAS-2	I^2 index was used; no method was used to explore the cause of heterogeneity	Egger's test
Nie et al. [27]	Prognostic importance of metabolic parameters (SUV, MTV) of ^{18}F-FDG PET in SCLC	MEDLINE, EMBASE, and Cochrane Library databases with English language studies only	Studies limited to SCLC patients who underwent pretreatment ^{18}F-FDG PET/CT and that was used as an initial imaging tool; MTV and/or TLG measurement of patients with SCLC; all patients received chemotherapy and/or radiotherapy; and articles that reported data relating to the impact of MTV or TLG on survival	Not reported	Cochrane's test and the I^2 statistic was used; sensitivity analysis was used to explore the cause of heterogeneity	Not reported
Piccardo et al. [28]	Head-to-head comparison between ^{18}F-FDOPA PET/CT and SSTR PET/CT in detecting intestinal NETs	PubMed, CENTRAL, Scopus and Web of Science, Embase were searched	Studies reporting a head-to-head comparison of ^{18}F-DOPA PET/CT and SSTR PET/CT in intestinal NETs patients	QUADAS-2	I^2 index was used. Threshold analysis using sROC. Subgroup analyses were done to explore the cause of heterogeneity	Egger's test
Rufini et al [29]	Diagnostic accuracy of ^{18}F-FDOPA PET and PET/CT in patients with NETs	PubMed/ MEDLINE, Embase and Scopus databases were search with no date or language limit	Studies with enough sample size (the exact number was not reported) on diagnostic accuracy of ^{18}F-FDOPA PET and PET/CT in NETs. Only studies with enough data to calculate diagnostic indices were included.	QUADAS-2	I^2 index was used. Threshold analysis using sROC. No other method was used to explore the reason of heterogeneity	Not reported
Shah et al. [30]	Diagnostic accuracy of Exendin-4 based PET/CT or SPECT/CT for insulinoma	Only PubMed and English language studies were searched	All studies on Exendin-4-based imaging for detecting insulinoma	Not reported	No true meta-analysis was done. Nothing on heterogeneity was reported	Not reported
Singh et al. [31]	To compare the role of SSTR PET or PET/CT in NETs with conventional imaging	MEDLINE, EMBASE, and Cochrane Database of Systematic Reviews databases were searched.	Studies comparing SSTR PET or PET/CT with conventional imaging in the initial diagnosis, staging and restaging, assessment of treatment response, and routine surveillance of NETs	QUADAS-2 only for diagnostic studies	I^2 index was used. Threshold analysis using sROC and bivariate meta-analysis. No other method was used	Not reported

		Only English studies were included			to explore the reason of heterogeneity	
Sun et al [32]	Diagnostic performance of ^{18}F-FDG PET in bone-bone marrow involvement in pediatric neuroblastoma	PubMed and Embase were searched without any date or language restriction	Studies included: (1) The main topic was the diagnostic accuracy of ^{18}F-FDG PET(CT) in the detection of bone or bone marrow involvement in pediatric NB patients; (2) Bone marrow biopsy as the gold standard; (3) sufficient data to reassess sensitivity and specificity	QUADAS-2	I2 index was used. Threshold analysis using sROC. Subgroup analyses were done to explore the cause of heterogeneity	Deeks' funnel plot asymmetry test
Treglia et al. [33]	Diagnostic performance of ^{18}F-FDOPA PET or PET/CT in detecting recurrent medullary thyroid carcinoma (MTC)	PubMed/ MEDLINE, Scopus, and Embase databases were searched. No language restriction	Studies or subsets in studies investigating the diagnostic performance of ^{18}F-FDOPA PET or PET/CT in patients with suspected recurrent MTC. Small sample studies were excluded (no number was reported)	QUADAS	I^2 index was used. Subgroup analyses were used to explore the reason of heterogeneity	Not reported
Treglia et al. [34]	Diagnostic performance of SSRT PET or PET/CT in patients with thoracic and/or gastroenteropancreatic (GEP) NETs	PubMed/MEDLINE, Scopus and Embase databases were searched without any date or language restriction	Diagnostic studies on SSRT PET or PET/CT in patients with thoracic and/or GEP NETs were eligible for inclusion (sample size > 8 cases)	QUADAS	I^2 index was used. Threshold analysis using sROC. Subgroup analyses were done to explore the cause of heterogeneity	Not reported
Treglia et al. [35]	Diagnostic performance of ^{18}F-FDOPA PET in patients with paraganglioma	PubMed/MEDLINE, Scopus and Embase databases were searched without any date or language restriction	Studies or subsets in studies investigating the diagnostic performance of ^{18}F-FDOPA PET or PET/CT in patients with paraganglioma. Small sample studies were excluded (no number was reported)	QUADAS	I^2 index was used. Threshold analysis using sROC. Subgroup analyses were done to explore the cause of heterogeneity	Not reported
Treglia et al. [36]	Diagnostic performance of ^{18}F-FDG PET and PET/CT in detecting recurrent medullary thyroid carcinoma (MTC)	PubMed/MEDLINE, Scopus and Embase databases were searched without any date or language restriction	Studies or subsets in studies investigating the diagnostic performance of ^{18}F-FDG PET or PET/CT in patients with recurrent/residual MTC were eligible for inclusion (sample size > 6 cases)	QUADAS	I^2 index was used. Subgroup analyses were done to explore the cause of heterogeneity	Not reported
Treglia et al. [37]	Diagnostic accuracy of ^{18}F-FDG PET in Merkel cell carcinoma (MCC)	PubMed/MEDLINE, Scopus databases were search without any date or language restriction	Studies or subsets in studies investigating the usefulness of ^{18}F-FDG PET or PET/CT in patients with MCC. Small sample studies were excluded (no number was reported)	The 2011 Oxford Center for Evidence-Based Medicine checklist for diagnostic studies	I^2 index was used. Threshold analysis using sROC. Subgroup analyses were done to explore the cause of heterogeneity	Funnel plots and Egger's test.
Treglia et al. [38]	Detection rate of SSRT PET in patients with recurrent MTC	PubMed/MEDLINE and Cochrane Library Databases were searched without date or language restriction	Studies or subsets in studies investigating the diagnostic performance of SSTR PET in patients with recurrent MTC. Case reports were excluded (no number was reported regarding sample size)	The 2011 Oxford Centre for Evidence-Based Medicine checklist for diagnostic studies	I2 index was used. Subgroup analyses were done to explore the cause of heterogeneity	Not reported
Xia et al. [39]	Comparison of diagnosing and staging accuracy of PET(/CT) and MIBG on patients with neuroblastoma (NB)	MEDLINE, EMBASE, Cochrane Library and CNKI databases without time limitation	Studies evaluating the performance of MIBG or PET(/CT) or both for examining neuroblastoma (sample size > 5 cases)	QUADAS-2	Cochrane Q test was used. Threshold analysis using sROC. Subgroup analyses were done to explore the cause of heterogeneity	Funnel plots were used

Yang et al. [40]	Diagnostic role of ^{68}Ga-DOTATOC and ^{68}Ga-DOTATATE PET in the diagnosis of NETs	Pubmed, Embase, and Scopus databases were searched only for English language studies	Papers investigating the diagnostic role of 68Ga-DOTATOC and 68Ga-DOTATATE PET in patients with NETs. Small sample studies were excluded (no number was reported)	QUADAS	I^2 index was used. Threshold analysis using sROC. Subgroup analyses were done to explore the cause of heterogeneity	Not reported
Zhu et al. [41]	Prognostic value of ^{18}F-FDG PET SUVmax of pretreatment primary lesions in SCLC	PubMed, EMBASE, the Cochrane library, and Web of Science were searched. Only English language studies.	(i) patients were histopathologically diagnosed with SCLC; (ii) the study was identified as a case-control or cohort study; (iii) at least one ^{18}F-FDG-PET/CT was performed before treatments, including chemotherapy, surgical therapy, and/or radiotherapy; (iv) at least one relevant prognostic factor was assessed, such as OS, PFS (v) hazard ratios (HRs) were available	Newcastle–Ottawa scale (NOS) checklist	Cochrane Q value and I^2 index were used. Subgroup analyses were done to explore the cause of heterogeneity	Begg's funnel test and Egger's test

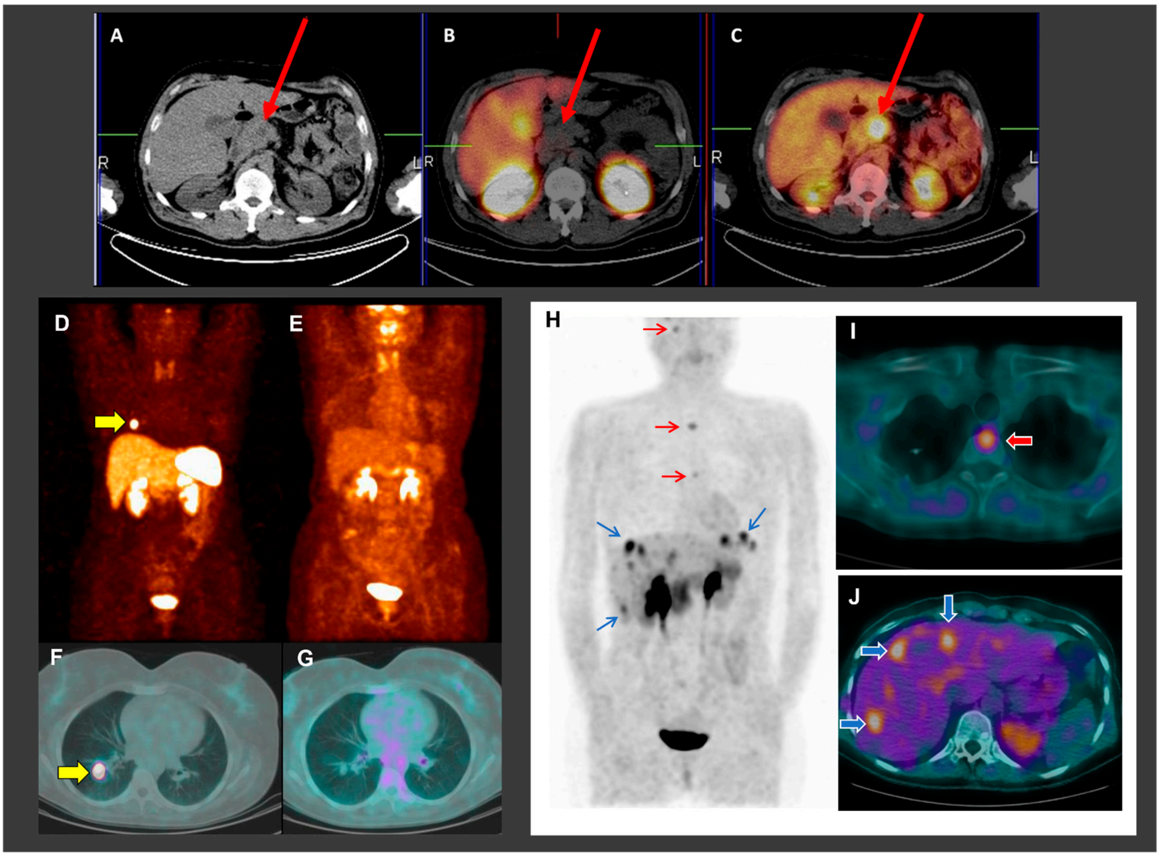

Figure S1. Case examples of the application of PET with different radiopharmaceuticals to detect neuroendocrine neoplasms. Axial computed tomography (CT) (**A**), hybrid somatostatin receptor imaging/CT (**B**) and fluorine-18 fluorodeoxyglucose positron emission tomography/CT (^{18}F-FDG PET/CT) (**C**) in a patient with high grade pancreatic neuroendocrine tumor (NET) showing increased ^{18}F-FDG uptake (increased glucose metabolism) and low uptake of radiolabeled somatostatin analogues (reduced expression of somatostatin receptors) due to its aggressive behavior. Somatostatin receptor PET (**D**), ^{18}F-FDG PET (**E**), axial somatostatin receptor PET/CT (**F**) and axial ^{18}F-FDG PET/CT images (**G**) in a patient with well-differentiated thoracic NET (typical bronchial carcinoid) showing increased uptake of radiolabeled somatostatin analogues (yellow arrows) due to increased somatostatin receptor expression and reduced ^{18}F-FDG uptake due to reduced glucose metabolism. ^{18}F-FDOPA PET (**H**) and axial ^{18}F-FDOPA PET/CT images (**I**,**J**) showing bone metastases (red arrows) and liver metastases (blue arrows) with increased radiopharmaceutical uptake in a patient with recurrent medullary thyroid carcinoma (with increased serum calcitonin levels).
